# Diagnostic Uncertainty in the Presentation of a Patient With 22q11.2 Deletion Syndrome: A Case Study

**DOI:** 10.1192/bjo.2026.11762

**Published:** 2026-06-30

**Authors:** Gabriel Augusto Araujo Vieira Marcque, John Carroll

**Affiliations:** Oxleas NHS Foundation Trust, London, United Kingdom

## Abstract

**Aims:**

22q11.2 deletion syndrome (22q11.2DS), the most common chromosomal microdeletion disorder, is associated with a heterogeneous presentation including congenital anomalies and later-onset conditions, such as cognitive delay, behavioural changes, psychiatric disorders, autoimmune diseases, and palatal, gastrointestinal, and renal abnormalities (1). Assessing mental health changes is often complex due to overlapping physical and psychiatric symptoms, contributing to diagnostic uncertainty. Distinguishing psychiatric relapse from challenging behaviour or physical health complications, such as pain or infection, requires careful assessment and highlights the importance of a multidisciplinary approach.

**Methods:**

Mr X is a 30-year-old male with mild intellectual disability secondary to 22q11.2DS and paranoid schizophrenia. He presented with several weeks of behavioural changes, including social withdrawal, fixed gaze episodes, “barking” vocalisations, erratic sleep, incongruent smiling, and attempts to leave his supported living accommodation. This was despite no apparent triggers and full adherence to risperidone. Investigations identified subclinical hypothyroidism, mild anaemia, suspected inflammatory bowel disease lost to follow-up, and recurrent otological pathology. Repeat neuroimaging was unremarkable. He had a history of positive response to temporary uptitration of risperidone during periods of relapse. There were clinical signs in keeping with psychotic relapse, however on this occasion uptitration of risperidone was not beneficial and resulted in upper extremity rigidity and increased drooling. Speech & Language Therapy input helped to clarify Mr X’s communication needs. Over time, episodes became shorter, less frequent & trigger-specific.Sleep improved with introduction of modified-release melatonin. Mr X has had a tympanoplasty, results are pending from an electroencephalogram, he is awaiting gastroenterology review and a sleep clinic assessment.

**Results:**

This case demonstrates the diagnostic complexity of psychiatric presentations in adults with 22q11.2DS, particularly in the context of intellectual disability and multimorbidity. Individuals with 22q11.2DS carry one of the highest known molecular risks for schizophrenia (prevalence 23.53–41%) (2), but behavioural, psychiatric and physical health factors increase the risk of diagnostic overshadowing. Adults with intellectual disability experience significant healthcare inequalities, with higher rates of avoidable mortality (3,4,5). For Mr X, proactive advocacy and coordinated multidisciplinary assessment were crucial in identifying organic contributors, supporting functional recovery, and avoiding inappropriate pharmacotherapy.

**Conclusion:**

Behavioural and psychiatric changes in adults with 22q11.2DS can reflect complex interactions between bio-psycho-social factors. Advocacy, multidisciplinary input and judicious psychotropic prescribing are essential for holistic management. Mr X’s case reinforces the importance of person-centred, integrated care in navigating diagnostic uncertainty and optimising outcomes for individuals with intellectual disability and 22q11.2DS.

